# Supplementary material for: α-Synuclein Aggregates in the Nigro-Striatal Dopaminergic Pathway Impair Fine Movement: Partial Reversal by the Adenosine A2A Receptor Antagonist
Source: Int J Mol Sci. 2023 Jan 10;24(2):1365. doi: 10.3390/ijms24021365 (PMC9866360; doi:10.3390/ijms24021365)
Supplement: Supplementary file 1 [file ijms-24-01365-s001.zip › ijms-2094765-supplementary.pdf]

Table S1. The numbers of mice used in all experiments.

Brain areas	Methods	Group	Mouse strain	Numbers of mice	Total numbers of mice	
DLS	Immunohistochemistry p-Syn	A53T	C57BL/6	3	A53T	12
		PBS	C57BL/6	3		
	Sunflower seed opening test	A53T	C57BL/6	12		
		PBS	C57BL/6	10	PBS	10
	Single-pellet reaching task	A53T	C57BL/6	7		
		PBS	C57BL/6	8		
	Locomotion	A53T	C57BL/6	12		
		PBS	C57BL/6	10		
SNc	Immunohistochemistry p-Syn	A53T	C57BL/6	3	A53T	19
		PBS	C57BL/6	3		
	TH-positive neurons	A53T	C57BL/6	3		
		PBS	C57BL/6	3		
	Sunflower seed opening test	A53T	C57BL/6	19		
		PBS	C57BL/6	18	PBS	18
	Single-pellet reaching task	A53T	C57BL/6	19		
		PBS	C57BL/6	17		
	Locomotion	A53T	C57BL/6	11		
		PBS	C57BL/6	18		
SNc	Sunflower seed opening test	A53T-αS KW6002	C57BL/6	9	A53T-αS KW6002	10
		A53T-αS Vehicle	C57BL/6	12		
	Single-pellet reaching task	A53T-αS KW6002	C57BL/6	10	A53T-αS Vehicle	12
		A53T-αS Vehicle	C57BL/6	12		
SNc	Immunohistochemistry h-Syn	A53T	PITX3-A53T transgenic mice	3	A53T	11
		nTg	WT littermates	3		
	TH-positive neurons	A53T	PITX3-A53T transgenic mice	5		
		nTg	WT littermates	5		
	Sunflower seed opening test	A53T	PITX3-A53T transgenic mice	11		
		nTg	WT littermates	17		
	Single-pellet reaching task	A53T	PITX3-A53T transgenic mice	9	nTg	19
		nTg	WT littermates	14		
	Locomotion	A53T	PITX3-A53T transgenic mice	11		
		nTg	WT littermates	19		
